# A student initiative to improve exposure in research – Dual benefit?

**DOI:** 10.1016/j.amsu.2020.06.033

**Published:** 2020-06-27

**Authors:** Marios Nicolaides, Kathrine Rallis, Pieter Jan Eyskens, Andreas Andreou, Funlayo Odejinmi, Apostolos Papalois, Michail Sideris

**Affiliations:** a Barts and the London School of Medicine and Dentistry, Queen Mary University of London, London, UK; b Whipps Cross University Hospital, Barts Health NHS Trust, London, UK; c Experimental Educational and Research Centre ELPEN, Athens, Greece; d European University Cyprus, Nicosia, Cyprus; e Women's Health Research Unit, Queen Mary University of London, London, UK

**Keywords:** Medical students, Dental students, Undergraduate medical education, Curriculum, Surveys and questionnaires

## Abstract

**Purpose:**

Despite the recent implementation of research-focused activities into undergraduate education, there is still a universal lack of offered exposure experienced by medical students. We organised an undergraduate research conference to explore students' views on research and evaluate the impact of the conference on participants' and organisers’ research skills and non-technical skills respectively.

**Methods:**

The conference was a student-led initiative which took place at a London medical school. Feedback from delegates was collected before and after the conference and aimed to evaluate previous experience and views in research, subjective assessment of relevant skills and the overall quality of the conference. Subjective change in organisers’ non-technical skill performance was also evaluated using an online questionnaire following the conference.

**Results:**

Forty-four students attended the conference, out of which only 3 (7.7%) have published in an international peer-reviewed journal. Finding a project supervisor was reported by most delegates as the biggest barrier in becoming involved in research. Delegates' study design (p = 0.041) and oral/poster presentation skills (p = 0.041) showed a statistically significant subjective improvement. A clear benefit in organisers’ subjective improvement in non-technical skill performance has been demonstrated. The conference was evaluated highly.

**Conclusion:**

There is need to address the barriers that medical and dental students face in the path to get involved in research. Our conference framework has demonstrated benefit to both delegates and organisers in improving their research skills and non-technical skills respectively. The conference, being highly appraised, lays the ground for such initiatives to be integrated in undergraduate medical and dental curricula.

## Highlights

• Less than 10% of students have published in peer-reviewed journals.
• Finding a project supervisor was reported as the biggest barrier in becoming involved in research.
• A student-led research conference can improve organisers' non-technical skills.
• A student-led research conference can improve delegates research and presentation skills.

## 1 Introduction

Living in an era of evidence-based medicine, the need for carrying out effective research has become a necessity for the advancement of the profession and, most importantly, patient care [1]. Thus, it is essential that medical professionals become engaged in research as early as in their undergraduate years; allowing enough time to hone fundamental skills in research methodology. In accord, the General Medical Council (GMC) considers the introduction and application of research principles in medical education a priority, as outlined in the ‘Outcomes for graduates 2018’ [2]. Moreover, the UK Foundation Programme Application System (FPAS) awards graduates with up to two points for peer-reviewed publications [3].

Despite the increasing recognition of research as a fundamental step towards the enhancement of healthcare provision, only a very small fraction of graduates follow a research-led career pathway [4]. This can be potentially attributed to the overcomplicated research governance and its limitations, discouraging students from carrying out their own original research, as identified by Robinson et al. [5] Nevertheless, efforts have been indeed made over the years to implement a variety of scholarly activities, including mentorship and scholarship programmes, acting as platforms between students and research personnel [6]. Successful attempts include initiatives encouraging students to undertake research activities and produce material suitable for scientific dissemination [7] Another example are Student-Selected-Components (SSCs), which are implemented in the curriculum of several UK medical schools and have as one of their aims to increase student exposure in research [8]; yet, this arguably happens at a very small scale.

Although implementations of research activities in medical schools' curricula have been evaluated before [9], there are no studies on the impact of student-led initiatives such as research conferences. Acknowledging the broad lack of student exposure to research, we organised a student-led undergraduate research conference for medicine and dentistry in an attempt to shift away from the typical restraint of such initiatives in grey areas of medical education. The objective of this study was to explore participants' views on research and evaluate the impact of the conference on participants' and organisers’ research skills and non-technical skills respectively.

## 2 Methods

Registration: This study is registered at Research Registry (registration number: researchregistry5664).

Conference model – the 360° approach: The conference was an inception of the Barts Academic and Research Society (BARS), a student-led society at Barts and the London School of Medicine and Dentistry, Queen Mary University of London. Students studying medicine and dentistry at UK universities were eligible to attend; places were made available online 3 months in advance. The main driving force for the organisation of the conference was a well-noted lack of student involvement in research at the University.

The structure of the conference, outlined in Table 1, is based on three main cores: key presentations, seminars and oral/poster presentations. Speaker and seminar selection were decided by the society committee with the support of expert faculty from the university and associated teaching hospitals.

**Table 1:** Outline of the conference programme.

Session	Description
Keynote Presentations	Delivered by leading consultants and academic researchers in their respective fields. Topics included: introduction to research, replacing animal research, assessment of surgeons through functional imaging and maxillofacial surgery. The common objective was to develop delegates' understanding of research in medicine and dentistry.
Seminars	Interactive teaching sessions held by consultants, junior doctors and senior medical students. Topics included: presentation skills, study design, steps to get involved in research and how to make the most of your research experience when applying for a job. The common objective was to develop delegates' fundamental research skills.
Delegate Presentations	Oral and poster presentations by undergraduate medical and dental students, following abstract submission and selection by a pre-formed committee. Presentations could be of any topic in medicine and dentistry and the best presentations received sponsored prizes. The common objective was to allow delegates to present their research and receive constructive feedback in a friendly environment.

Feedback Collection: Feedback from delegates was collected before (Appendix 1) and after (Appendix 2) the conference; the former using printed questionnaires and the latter using an online platform. Questionnaires consisted of a concoction of binary, likert-scale and short-answer questions, which aimed to evaluate students’ previous experience and views on research, subjective assessment of relevant skills, and the overall quality of the conference.

Subjective change in organisers’ (student committee) non-technical skill performance was also evaluated using an online questionnaire following the conference (Appendix 3).

Statistics: Data were transferred to a digital spreadsheet and were analysed using IBM SPSS Statistics for Windows, Version 23. Statistical tests used were the Mann-Whitney *U* test, a non-parametric unpaired sample *t*-test for comparing pre- and post-conference likert-scale responses; and the chi-square test for binary yes/no responses appropriate for unmatched sample data.

## 3 Results

**Fig. 1:**
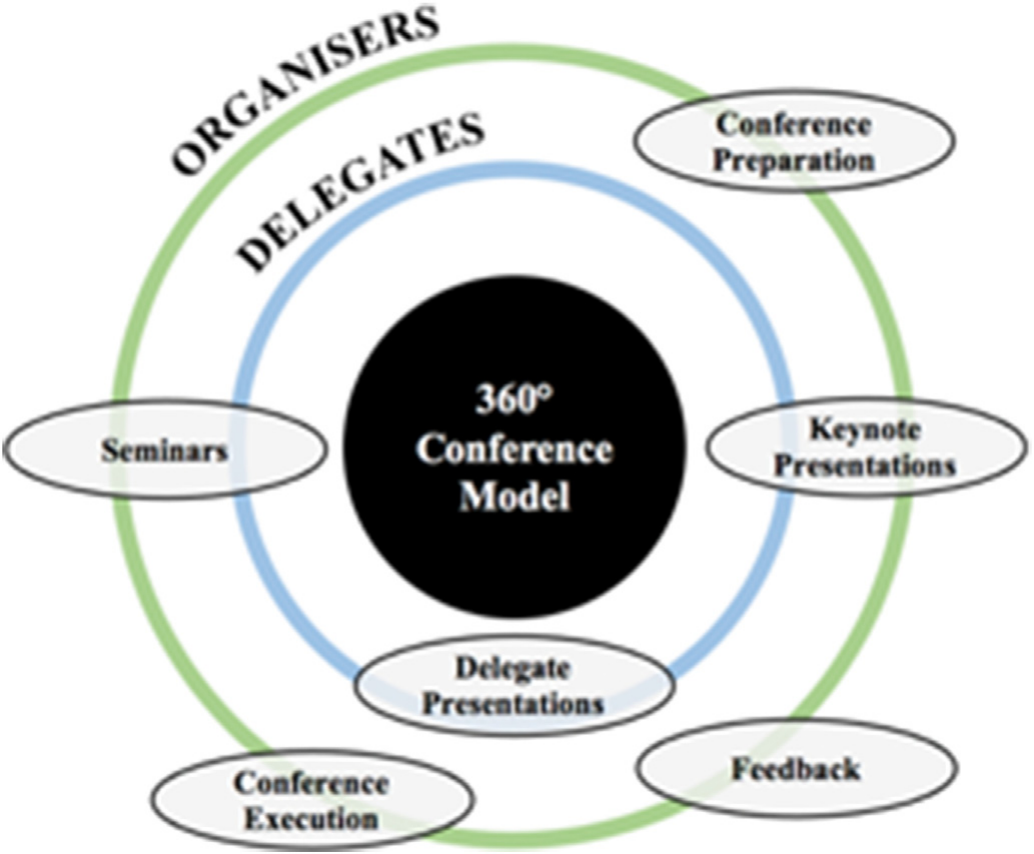
Delegates and organisers' year of study, not including intercalation.

Delegate demographics and exposure to research: Forty-four students attended the conference, out of which 39 (88.6%) completed the pre-conference questionnaire and 43 (97.7%) completed the post-conference questionnaire. Most participants were medical students (38 out of 39 respondents) and the median age was 21.00 (IQR = 2). The majority of delegates were in the second year of their studies (36.7%), whereas there were no students in the final year (Fig. 1). Noteworthily, only 3 students (7.7%) have published in an international peer-reviewed journal and 17 students (43.6%) did not receive any research skill training (Supplementary Table 1).

Delegates' views on research: Even though there was no significant difference between delegates' views on the importance of research pre- and post-conference, in both instances the ‘importance of being involved in research as a student’ scored less highly than the ‘importance of research in general’ (Supplementary Table 2). Furthermore, delegates' responses collectively demonstrate an overwhelming interest in being involved in research (Supplementary Table 3).

Following the conference, there was an increase in delegate awareness of points awarded for publications in the UK FPAS and of the GMC emphasis on integrating scientific methods and medical research in decision-making for care. This was statistically significant in both instances, p = 0.009 and p = 0.039 respectively.

There is a unanimous agreement in the post-conference questionnaire that students face barriers in becoming involved in research (Supplementary Table 3). Finding a project supervisor was reported by most delegates as the biggest barrier in becoming involved in research (20 out of 39 in pre-, 19 out of 43 in post-conference questionnaire). Furthermore, more participants recognised lack of expertise as a barrier following the conference (5 out of 39 in pre-, 17 out of 43 in post-conference questionnaire) (Supplementary Table 4).

Delegates' subjective assessment of research skills: Delegates’ subjective assessment of their research skills in different domains are outlined in Table 2. The only domains that showed a statistically significant increase after the conference were study design (p = 0.041) and oral/poster presentation skills (p = 0.041).

**Table 2:** Delegate subjective assessment of skills pre- and post-conference.

	Pre-Conference							Post-Conference						
Response	Searching the Literature	Reading a research article effectively	Study design	Data analysis	Writing a manuscript for publication	Presenting your own research in an oral or poster presentation	Critically appraising a research article	Searching the Literature	Reading a research article effectively	Study design	Data analysis	Writing a manuscript for publication	Presenting your own research in an oral or poster presentation	Critically appraising a research article
**1 – Awful**	0	0	5	4	11	4	1	0	0	1	0	1	0	0
**2**	6	5	10	8	7	6	7	0	0	8	8	11	1	6
**3**	19	21	18	19	13	15	22	22	22	22	25	22	20	19
**4**	10	9	6	8	8	10	6	20	20	12	10	9	18	15
**5 –Excellent**	4	4	0	0	0	4	3	1	1	0	0	0	4	3
**Median response (IQR)**	3 (3–4)	3 (3–4)	3 (2–3)	3 (2–3)	3 (1–3)	3 (2–4)	3 (3–3)	3 (3–4)	3 (3–4)	3 (3–4)	3 (3–3)	3 (2–3)	4 (3–4)	3 (3–4)

Organisers subjective assessment of non-technical skills: All conference-organisers (n = 10) completed a post-conference questionnaire; including 8 (80%) medical students and 2 (20%) dental students. Fig. 1 demonstrates the organisers’ year of study.

Table 3 demonstrates a clear benefit in organisers’ subjective change in non-technical skill performance with median Likert-scale scores of 4 or 5 across all domains. Significant results were observed for organisers appreciation of the importance of role assignment within the team with 9 out of 10 organisers strongly agreeing with this statement. Confidence in organising another conference in the future received the second highest rating (8 out of 10 strongly agree, 2 out of 10 agree), while improvement in teamwork skills, personal development benefit, and professional development/career progression benefit were also scored highly with median Likert-scale response of 5 across all.

**Table 3:** Organisers’ subjective change in non-technical skill performance.

**Response**	I have improved my communication skills	I have improved my teamwork skills	I have improved my organisational skills	I have improved my decision-making skills	I appreciate the importance of having specific roles assigned within the team	My involvement in the organising committee has been beneficial for my personal development	My involvement in the organising committee has been beneficial for my professional development/career progression	I am more confident in organising another conference in the future
**1–Strongly disagree**	0	0	0	0	0	0	0	0
**2**	0	0	0	0	0	0	0	0
**3**	1	0	1	3	0	1	2	0
**4**	6	4	7	4	1	2	2	2
**5–Strongly agree**	3	6	2	3	9	7	6	8
**Median response (IQR)**	4 (4–5)	5 (4–5)	4 (4–4.25)	4 (3–5)	5 (5–5)	5 (3.75–5)	5 (4.75–5)	5 (4–5)

Conference model evaluation and impact: The conference was evaluated highly by both delegates and organisers. Highest rating was observed for organisers' satisfaction of the conference outcome (7 out of 10 strongly agree, 3 out of 10 agree) (Table 4). As for the delegates’ evaluations, most agreed they would recommend this conference to a colleague (18 strongly agree and 16 agree out of 43). Moreover, most delegates awarded high Likert-scale ratings (median = 4) for conference assessment domains including relevance and engagingness of content, appropriate level of delivery, overall conference satisfaction, and importance of such conferences in undergraduate medical education.

**Table 4:** Conference evaluation and impact according to delegates.

	Delegates									Organisers	
Response											
	The conference content was relevant and engaging	The content was delivered at an appropriate level	I can appreciate the skills required to get involved in research	I am more motivated to get involved in research and publish a paper	I feel more confident in engaging with a research department at my university	Such conferences are essential in undergraduate medical/dental education	I am very satisfied with the conference overall	I will recommend this conference to my colleagues	I am satisfied with the outcome of the conference		
**1–Strongly disagree**	0	0	0	0	0	0	0	0	0		
**2**	0	0	0	0	0	1	0	0	0		
**3**	6	4	3	5	10	6	6	9	0		
**4**	29	27	24	17	18	19	24	16	3		
**5–Strongly agree**	8	12	16	21	15	17	13	18	7		
**Median response (IQR)**	4 (4–4)	4 (4–5)	4 (4–5)	4 (4–5)	4 (4–5)	4 (4–5)	4 (4–5)	4 (4–5)	5 (4–5)		

Following the conference, delegates were highly motivated to get involved in research and publish a paper (21 out of 43 strongly agree, 17 out of 43 agree). Furthermore, delegates became more aware of the skills required to get involved in research, and more confident in engaging with a research department at university (median Likert-scale rating of 4).

## 4 Discussion

**Fig. 2:**
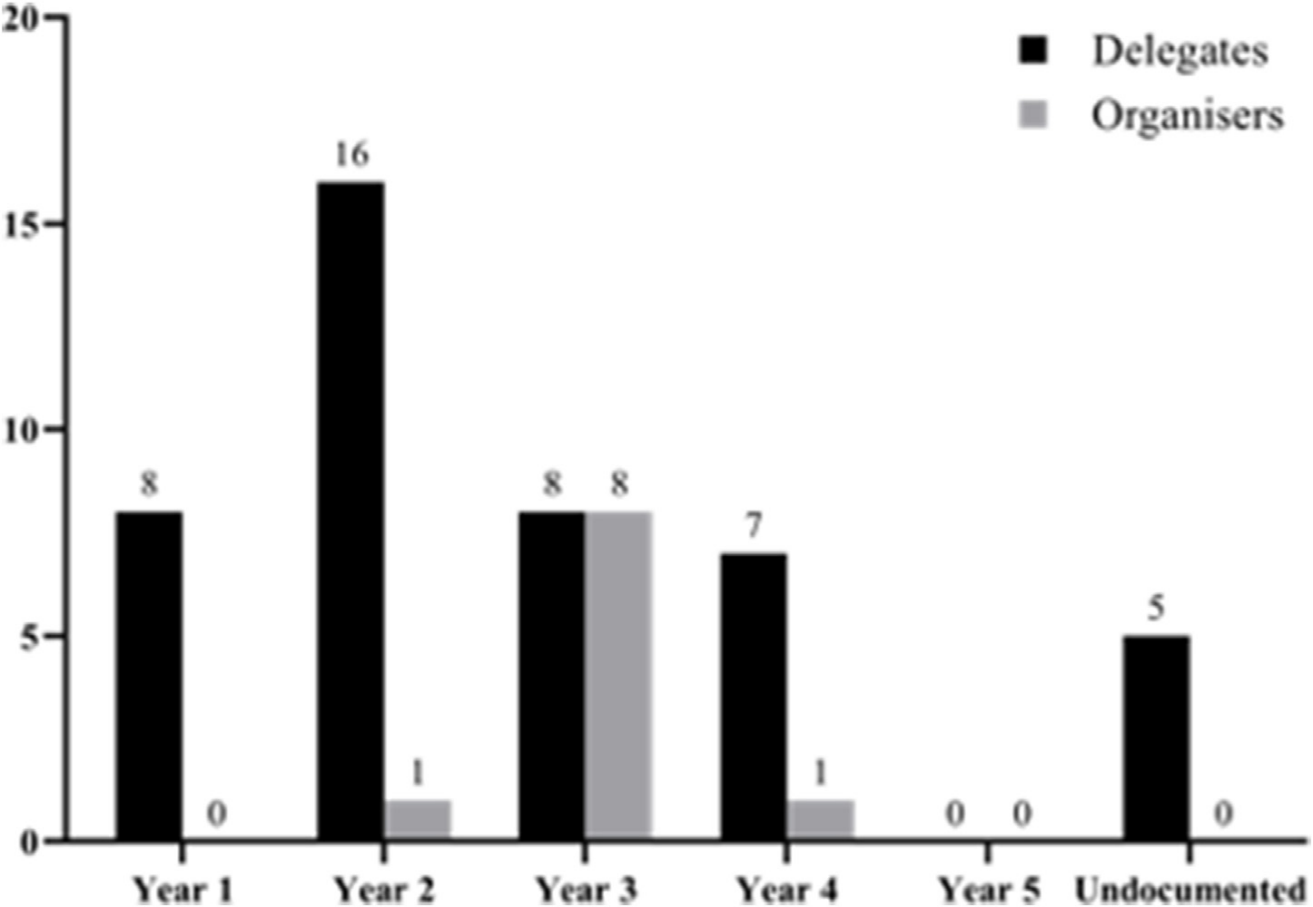
Our 360-degree conference model structure, yielding benefits to both delegates and organisers.

The 360° conference model: Our conference model (Fig. 2) takes a promising approach of exploring delegate views and improving delegates' and student-organisers’ research and non-technical skills, respectively. Considering the limited resources, faculty and amount of time available in medical education today, activities that can benefit a higher number of students should be utilised. Thus, a 360-degree approach based on peer-teaching methods that yields profits for both organisers and delegates, seems to be an effective approach for tackling the aforementioned challenges. Yet, further refinement is required to establish its efficacy and adaptability.

Extensive evidence in the literature has deemed peer-teaching methods to be beneficial in undergraduate medical education [[10], [11], [12], [13]]. Yu et al. have concluded that “not only it is comparable to conventional teaching, but there is also evidence to suggest that participating student-teachers benefit academically and professionally” [13]. Additionally, our conference has allowed for other non-technical skills and attitudes to prosper, including teamwork, communication, decision-making and organisational skills. Furthermore, the combination of keynote presentations and small-group teaching has been previously proved to be an effective method of training for medical students [14].

The fact that the conference was highly appraised suggests that it can be advantageous in developing a positive attitude towards research, with a resultant increase in delegates' confidence and competence (Table 4). Moreover, the high conference satisfaction ratings by both delegates and organisers in conjunction with the delegates’ likelihood to recommend this conference to a colleague and the high rating for the essentialness of such conferences in undergraduate medical education, advocate for further use of such peer-teaching conference initiatives and testify to their potential for success.

Delegate views on research: The questionnaire findings come in line with our initial speculations that there is a profound lack of involvement in research at the undergraduate level in our institution. This is particularly supported by the fact that only a fraction of participants (7.7%) had previously published in an international peer-reviewed journal. More importantly, this cannot be attributed to lack of interest, since prior to the conference the majority of students (94.9%) were indeed interested in being involved in a research project. Hence, we can arguably assume that students are impeded from getting involved in research. This is further supported by the post-conference unanimous agreement on the existence of barriers, with the most profound being finding a project or supervisor and lack of expertise. A potential solution to the former is the introduction of an online platform, allowing academics to post vacancies in their research teams and students then applying by uploading a short personal statement and curriculum vitae.

Although medical students seem keen to publish a research study, most report that their undergraduate curriculum fails to provide the appropriate training (43.6% of delegates stated that they did not receive any research skills training at all); consequently, demonstrating lack of knowledge. Such gaps can be bridged by developing more robust research-methodology modules, either as part of the medical syllabus or as extra-curricular activities. The importance of such practices is highlighted by both the GMC “Outcomes for Graduates” 2018 and by the recognition of publication in UK FPAS. Even though basic lecture-based teaching on research methodology is already implemented in the undergraduate curriculum of our institution, there is no formal training on how students can become involved in research projects; likely, this seems to be the case at other UK medical schools as well. Furthermore, the fact that delegates scored the importance of being involved in research as a student lower than the importance of research in general may reflect a lack of emphasis being placed on such involvement. Potential solutions to these issues include introduction of stand-alone interactive workshops, similar to the ones set up in our conference model.

Dual benefit: The results of the study reflect a benefit to both delegates and organisers. As noted, there was a subjective improvement of several delegate research skills (study design and research presentation). The use of interactive teaching techniques to deliver the workshops positively correlates to a statistically significant improvement in those skills. Future research should focus on how such techniques can be utilised in similar conference workshops, within medical degree modules or even as stand-alone short courses. The fact that other measured research skills did not show any significant improvement can be attributed to time constraints created by having the conference on a single day.

The student-organisers of the conference also seem to have benefited from this activity. As marked by the questionnaires, there was an observed improvement in all non-technical skills of the organisers. Such skills are essential to nurture healthcare professionals who can work competently in modern healthcare systems and reduce medical errors. Although there is increased emphasis on their importance, there is still a need for more structured implementation within undergraduate training [15]. Moreover, a follow-up assessment of both delegates and organisers would be beneficial to evaluate long-term retention of taught skills.

## 5 Limitations

Our conclusions have been drawn from a relatively small sample size and only a limited amount of time was available for skill training. Additionally, evaluation of any improvement was subjective. Finally, even though the conference attracted delegates from various UK medical schools, the fact still remains that it took place in a single institution. To evaluate this model further, future studies should explore and evaluate the integrity of such approach at other universities with objective, validated methods of assessment.

## 6 Conclusions

Upon recognising the views of delegates and organisers in regard to research in general, it can be concluded that there is need to address the barriers that medical and dental students face in the path to getting involved in research. Furthermore, our results suggest that students of our institution found this type of conference framework valuable, irrespective of them being delegates or organisers, in gaining essential research and non-technical skills. Lastly, the conference has been a positive learning experience for all students and lays the ground for such initiatives to be integrated in the undergraduate medical curricula.

## Provenance and peer review

Not commissioned, externally peer reviewed.

## Ethical approval

This study did not require ethical approval and it was held as part of a national conference.

## Sources of funding

No funding received for this manuscript.

## Author contribution

Conceptualization: MN MS AP FO.

Data curation: MN AA PJE KR.

Formal analysis: MN KR.

Methodology: MN.

Project administration: MN.

Visualization: MN.

Writing – original draft: MN AA PJE KR.

Writing – review & editing: MN KR PJE AA MS FO.

## Registration of research studies

Registration: This study is registered at Research Registry (registration number: researchregistry5664).

https://www.researchregistry.com/browse-theregistry#home/?view_2_search = researchregistry5664&view_2_page = 1.

## Guarantor

Michail Sideris is the guarantor of this study.

## Consent

Not required.

## Declaration of competing interest

None declared.

## Footnotes

Appendix A Supplementary data to this article can be found online at https://doi.org/10.1016/j.amsu.2020.06.033.
