# Supplementary material for: A student initiative to improve exposure in research – Dual benefit?
Source: Ann Med Surg (Lond). 2020 Jun 27;56:211–6. doi: 10.1016/j.amsu.2020.06.033 (PMC7355375; doi:10.1016/j.amsu.2020.06.033)
Supplement: Multimedia component 1 [file mmc1.docx]

**Supplementary table 1** Delegate research experience prior to the conference.

| Response | Have you ever been involved in a research project? | Have you published a paper in an international peer-reviewed journal? | Have you had any kind of research skill training (e.g. critical appraisal, how to read a paper, how to design a study, how to search the literature)? | Have you ever presented original research at an international conference? |
| --- | --- | --- | --- | --- |
| Yes | 18 | 3 | 22 | 3 |
| No | 21 | 36 | 17 | 36 |
| Not-documented | 5 | 5 | 5 | 5 |

**Supplementary table 2** Comparison of pre- versus post-conference ratings of the importance of research in medicine and dentistry and the importance of student involvement in research.

| Response | Pre-Conference | | Post-Conference | |
| --- | --- | --- | --- | --- |
|  | **How would you rate the importance of research in medicine/dentistry?** | **How would you rate the importance of being involved in research as a medical/dental student?** | **How would you rate the importance of research in medicine/dentistry?** | **How would you rate the importance of being involved in research as a medical/dental student?** |
| 1 – Not important at all | 0 | 0 | 0 | 0 |
| 2 | 0 | 0 | 0 | 0 |
| 3 | 1 | 5 | 0 | 6 |
| 4 | 7 | 19 | 7 | 23 |
| 5 – Very important | 31 | 15 | 36 | 14 |
| Median response (IQR) | 5 (5-5) | 4 (4-5) | 5 (5-5) | 4 (4-5) |

**Supplementary table 3** Comparison of delegates’ interest in becoming involved in research and their perceived presence of barriers pre- versus post-conference.

|  | **Pre-Conference** | | **Post-Conference** | |
| --- | --- | --- | --- | --- |
|  | **Yes** | **No** | **Yes** | **No** |
| **Would you be interested in being involved in a research project?** | 37 | 2 | 42 | 1 |
| **Do you think that medical/dental students face any barriers in getting involved in research?** | 36 | 3 | 43 | 0 |

**Supplementary table 4** Comparison of delegates’ interest in becoming involved in research and their perceived presence of barriers pre- versus post-conference.

| Response | If yes, which do you think is the biggest barrier? | |
| --- | --- | --- |
|  | **Pre-Conference** | **Post-Conference** |
| Finding a project or supervisor | 20 | 19 |
| Lack of free time | 7 | 4 |
| Lack of expertise | 5 | 17 |
| Lack of skills | 3 | 2 |
| Other (funding) | 1 | 1 |
| N/A | 3 | 0 |
