# Supplementary material for: A student initiative to improve exposure in research – Dual benefit?
Source: Ann Med Surg (Lond). 2020 Jun 27;56:211–6. doi: 10.1016/j.amsu.2020.06.033 (PMC7355375; doi:10.1016/j.amsu.2020.06.033)
Supplement: Multimedia component 3 [file mmc3.pdf]

Delegate Questionnaire

Undergraduate Research Conference for Medicine and Dentistry - 15 December 2018
Whitechapel Campus, Barts and the London School of Medicine and Dentistry

Please fill out this questionnaire to help us improve. Questionnaire data might be used for research purposes. Thank you.

1. **Degree**
   Mark only one oval.
   - ( ) Medicine
   - ( ) Dentistry

2. **Year of Study (Do NOT include years of intercalation/other degrees)**
   Mark only one oval.
   - ( ) 1
   - ( ) 2
   - ( ) 3
   - ( ) 4
   - ( ) 5

3. **Medical School**

   ____

4. **Age**

   ____

5. **Previous Degree**
   Check all that apply.
   - [ ] No previous degree
   - [ ] BSc
   - [ ] iBSc
   - [ ] MSc
   - [ ] Other (please specify): _______________

Previous experience

6. **Have you ever been involved in a research project?**
   Mark only one oval.
   - ( ) Yes
   - ( ) No

7. **Have you published a paper in an international peer-reviewed journal?**
Mark only one oval.

- ⬭ Yes
- ⬭ No

8. **If yes, how many international peer-reviewed publications do you have?**

____

9. **Have you had any kind of research skill training (e.g. critical appraisal, how to read a paper, how to design a study, how to search the literature)?**
Mark only one oval.

- ⬭ Yes
- ⬭ No

10. **If yes, please specify (e.g. critical appraisal workshop and study design workshop)**

____

11. **Have you ever presented original research at an international conference?**
Mark only one oval.

- ⬭ Yes
- ⬭ No

12. **If you have intercalated, do you think that this has helped you into getting involved in research?**
Mark only one oval.

- ⬭ I have not intercalated
- ⬭ Yes, it has helped
- ⬭ No, it has not helped

Views on Research

13. **How would you rate the importance of research in medicine/dentistry?**
Mark only one oval.

	1	2	3	4	5	
Not important at all	⬭	⬭	⬭	⬭	⬭	Very important

14. **How would you rate the importance of being involved in research as a medical/dental student?**
Mark only one oval.

	1	2	3	4	5	
Not important at all	◯	◯	◯	◯	◯	Very important

15. **Would you be interested in being involved in a research project?**
Mark only one oval.

◯ Yes
◯ No
◯ Already involved

16. **Do you think that medical/dental students face any barriers in getting involved in research?**
Mark only one oval.

◯ Yes
◯ No

17. **If yes, which do you think is the biggest barrier?**
Mark only one oval.

◯ Lack of free time
◯ Lack of skills
◯ Finding a project or supervisor
◯ Lack of expertise
◯ Other - please specify ____________________

18. **Are you aware that publications can get a maximum of two points in the application score of the UK Foundation Programme?**
Mark only one oval.

◯ Yes
◯ No

19. **Are you aware that the GMC 'Outcomes for Graduates 2018' require medical school graduates to be able to apply scientific methods and approaches to medical research and integrate these with a range of sources of information used to make decisions for care?**
Mark only one oval.

◯ Yes
◯ No

Skills

20. **How would you rate your skills in: Searching the Literature?**
Mark only one oval.

	1	2	3	4	5	
Awful	◯	◯	◯	◯	◯	Excellent

21. **How would you rate your skills in: Reading a research article effectively?**
Mark only one oval.

	1	2	3	4	5	
Awful	◯	◯	◯	◯	◯	Excellent

22. **How would you rate your skills in: Study design?**
Mark only one oval.

	1	2	3	4	5	
Awful	◯	◯	◯	◯	◯	Excellent

23. **How would you rate your skills in: Data analysis?**
Mark only one oval.

	1	2	3	4	5	
Awful	◯	◯	◯	◯	◯	Excellent

24. **How would you rate your skills in: Writing a manuscript for publication?**
Mark only one oval.

	1	2	3	4	5	
Awful	◯	◯	◯	◯	◯	Excellent

25. **How would you rate your skills in: Presenting your own research in an oral or poster presentation?**
Mark only one oval.

	1	2	3	4	5	
Awful	◯	◯	◯	◯	◯	Excellent

26. **How would you rate your skills in: Critically appraising a research article?**
Mark only one oval.

	1	2	3	4	5	
Awful	◯	◯	◯	◯	◯	Excellent

Powered by
Google Forms
