# Supplementary material for: A student initiative to improve exposure in research – Dual benefit?
Source: Ann Med Surg (Lond). 2020 Jun 27;56:211–6. doi: 10.1016/j.amsu.2020.06.033 (PMC7355375; doi:10.1016/j.amsu.2020.06.033)
Supplement: Multimedia component 4 [file mmc4.pdf]

Organising Questionnaire

Undergraduate Research Conference for Medicine and Dentistry - 15 December 2018
Whitechapel Campus, Barts and the London School of Medicine and Dentistry

1. **Degree**
   Mark only one oval.

   ◯ Medicine
   ◯ Dentistry

2. **Year of Study (Do NOT include years of intercalation/other degrees)**
   Mark only one oval.

   ◯ 1
   ◯ 2
   ◯ 3
   ◯ 4
   ◯ 5

3. **Age**

   ____

4. **Previous Degree**
   Check all that apply.

   ☐ No previous degree
   ☐ BSc
   ☐ iBSc
   ☐ MSc
   ☐ Other (please specify): _______________

Previous experience

5. **Have you ever been involved in the organisation of another conference before?**
   Mark only one oval.

   ◯ Yes
   ◯ No

6. **If yes, how many conferences have you organised before?**

   ____

Views on Research

7. **How would you rate the importance of research in medicine/dentistry?**
   Mark only one oval.

	1	2	3	4	5	
Not important at all	◯	◯	◯	◯	◯	Very important

8. **How would you rate the importance of being involved in research as a medical/dental student?**
   Mark only one oval.

	1	2	3	4	5	
Not important at all	◯	◯	◯	◯	◯	Very important

9. **Would you be interested in being involved in a research project?**
   Mark only one oval.

   - ◯ Yes
   - ◯ No
   - ◯ Already involved

10. **Do you think that medical/dental students face any barriers in getting involved in research?**
    Mark only one oval.

    - ◯ Yes
    - ◯ No

11. **If yes, which do you think is the biggest barrier?**
    Mark only one oval.

    - ◯ Lack of free time
    - ◯ Lack of skills
    - ◯ Finding a project or supervisor
    - ◯ Lack of expertise
    - ◯ Other - please specify ______________________

12. **Are you aware that publications can get a maximum of two points in the application score of the UK Foundation Programme?**
    Mark only one oval.

    - ◯ Yes
    - ◯ No

13. **Are you aware that the GMC 'Outcomes for Graduates 2018' require medical school graduates to be able to apply scientific methods and approaches to medical research and integrate these with a range of sources of information used to make decisions for care?**

Mark only one oval.

- ◯ Yes
- ◯ No

Skills

To what extend do you agree with the following statements, after being part of the organising committee

14. **I have improved my communication skills**

Mark only one oval.

	1	2	3	4	5	
Strongly disagree	◯	◯	◯	◯	◯	Strongly agree

15. **I have improved my teamwork skills**

Mark only one oval.

	1	2	3	4	5	
Strongly disagree	◯	◯	◯	◯	◯	Strongly agree

16. **I have improved my organisational skills**

Mark only one oval.

	1	2	3	4	5	
Strongly disagree	◯	◯	◯	◯	◯	Strongly agree

17. **I have improved my decision-making skills**

Mark only one oval.

	1	2	3	4	5	
Strongly disagree	◯	◯	◯	◯	◯	Strongly agree

18. **I appreciate the importance of having specific roles assigned within the team**

Mark only one oval.

	1	2	3	4	5	
Strongly disagree	◯	◯	◯	◯	◯	Strongly agree

19. **My involvement in the organising committee has been beneficial for my personal development**

Mark only one oval.

	1	2	3	4	5	
Strongly disagree	◯	◯	◯	◯	◯	Strongly agree

20. **My involvement in the organising committee has been beneficial for my professional development/career progression**

Mark only one oval.

	1	2	3	4	5	
Strongly disagree	◯	◯	◯	◯	◯	Strongly agree

21. **I am more confident in organising another conference in the future**

Mark only one oval.

	1	2	3	4	5	
Strongly disagree	◯	◯	◯	◯	◯	Strongly agree

22. **I am satisfied with the outcome of the conference**

Mark only one oval.

	1	2	3	4	5	
Strongly disagree	◯	◯	◯	◯	◯	Strongly agree

Powered by
Google Forms
