# Supplementary material for: A student initiative to improve exposure in research – Dual benefit?
Source: Ann Med Surg (Lond). 2020 Jun 27;56:211–6. doi: 10.1016/j.amsu.2020.06.033 (PMC7355375; doi:10.1016/j.amsu.2020.06.033)
Supplement: Multimedia component 5 [file mmc5.pdf]

Delegate Questionnaire

Undergraduate Research Conference for Medicine and Dentistry - 15 December 2018
Whitechapel Campus, Barts and the London School of Medicine and Dentistry

Please fill out this questionnaire to help us improve. Questionnaire data might be used for research purposes. Certificates of attendance are provided once you have submitted your response. Thank you.

* Required

Views on Research

1. **How would you rate the importance of research in medicine/dentistry?** *
   Mark only one oval.

	1	2	3	4	5	
Not important at all	◯	◯	◯	◯	◯	Very important

2. **How would you rate the importance of being involved in research as a medical/dental student?** *
   Mark only one oval.

	1	2	3	4	5	
Not important at all	◯	◯	◯	◯	◯	Very important

3. **Would you be interested in being involved in a research project?** *
   Mark only one oval.

   ◯ Yes
   ◯ No
   ◯ Already involved

4. **Do you think that medical/dental students face any barriers in getting involved in research?** *
   Mark only one oval.

   ◯ Yes
   ◯ No

5. **If yes, which do you think is the biggest barrier?** *
Mark only one oval.

- Lack of free time
- Lack of skills
- Finding a project or supervisor
- Lack of expertise
- Other - please specify ____________________

6. **Are you aware that publications can get a maximum of two points in the application score of the UK Foundation Programme?** *
Mark only one oval.

- Yes
- No

7. **Are you aware that the GMC 'Outcomes for Graduates 2018' require medical school graduates to be able to apply scientific methods and approaches to medical research and integrate these with a range of sources of information used to make decisions for care?** *
Mark only one oval.

- Yes
- No

Skills

8. **How would you rate your skills in: Searching the Literature?** *
Mark only one oval.

	1	2	3	4	5	
Awful						Excellent

9. **How would you rate your skills in: Reading a research article effectively?** *
Mark only one oval.

	1	2	3	4	5	
Awful						Excellent

10. **How would you rate your skills in: Study design?** *
Mark only one oval.

	1	2	3	4	5	
Awful						Excellent

11. **How would you rate your skills in: Data analysis?** *
Mark only one oval.

	1	2	3	4	5	
Awful	◯	◯	◯	◯	◯	Excellent

12. **How would you rate your skills in: Writing a manuscript for publication?** *
Mark only one oval.

	1	2	3	4	5	
Awful	◯	◯	◯	◯	◯	Excellent

13. **How would you rate your skills in: Presenting your own research in an oral or poster presentation?** *
Mark only one oval.

	1	2	3	4	5	
Awful	◯	◯	◯	◯	◯	Excellent

14. **How would you rate your skills in: Critically appraising a research article?** *
Mark only one oval.

	1	2	3	4	5	
Awful	◯	◯	◯	◯	◯	Excellent

Conference

How much do you agree with the following statements after the conference?

15. **The conference content was relevant and engaging** *
Mark only one oval.

	1	2	3	4	5	
Strongly disagree	◯	◯	◯	◯	◯	Strongly agree

16. **The content was delivered at an appropriate level.** *
Mark only one oval.

	1	2	3	4	5	
Strongly disagree	◯	◯	◯	◯	◯	Strongly agree

17. **I can appreciate the skills required to get involved in research.** *
Mark only one oval.

	1	2	3	4	5	
Strongly disagree	⬭	⬭	⬭	⬭	⬭	Strongly agree

18. **I am more motivated to get involved in research and publish a paper.** *
Mark only one oval.

	1	2	3	4	5	
Strongly disagree	⬭	⬭	⬭	⬭	⬭	Strongly agree

19. **I feel more confident in engaging with a research department at my university.** *
Mark only one oval.

	1	2	3	4	5	
Strongly disagree	⬭	⬭	⬭	⬭	⬭	Strongly agree

20. **Such conferences are essential in undergraduate medical/dental education.** *
Mark only one oval.

	1	2	3	4	5	
Strongly disagree	⬭	⬭	⬭	⬭	⬭	Strongly agree

21. **I am very satisfied with the conference overall.** *
Mark only one oval.

	1	2	3	4	5	
Strongly disagree	⬭	⬭	⬭	⬭	⬭	Strongly agree

22. **I will recommend this conference to my colleagues.** *
Mark only one oval.

	1	2	3	4	5	
Strongly disagree	⬭	⬭	⬭	⬭	⬭	Strongly agree

Suggestions

23. **Is there anything else you would like to add?**

Powered by
Google Forms
